# Spinal electrical stimulation to improve sympathetic autonomic functions needed for movement and exercise after spinal cord injury: a scoping clinical review

**DOI:** 10.1152/jn.00205.2022

**Published:** 2022-07-27

**Authors:** Sarah Flett, Juanita Garcia, Kristine C. Cowley

**Affiliations:** Department of Physiology and Pathophysiology, Rady Faculty of Health Sciences, University of Manitoba, Winnipeg, Manitoba, Canada

**Keywords:** cardiovascular, exercise, paraplegia, somato-sympathetic integration, tetraplegia

## Abstract

Spinal cord injury (SCI) results in sensory, motor, and autonomic dysfunction. Obesity, cardiovascular disease, and metabolic disease are highly prevalent after SCI. Although inadequate voluntary activation of skeletal muscle contributes, it is absent or inadequate activation of thoracic spinal sympathetic neural circuitry and suboptimal activation of homeostatic (cardiovascular and temperature) and metabolic support systems that truly limits exercise capacity, particularly for those with cervical SCI. Thus, when electrical spinal cord stimulation (SCS) studies aimed at improving motor functions began mentioning effects on exercise-related autonomic functions, a potential new area of clinical application appeared. To survey this new area of potential benefit, we performed a systematic scoping review of clinical SCS studies involving these spinally mediated autonomic functions. Nineteen studies were included, 8 used transcutaneous and 11 used epidural SCS. Improvements in blood pressure regulation at rest or in response to orthostatic challenge were investigated most systematically, whereas reports of improved temperature regulation, whole body metabolism, and peak exercise performance were mainly anecdotal. Effective stimulation locations and parameters varied between studies, suggesting multiple stimulation parameters and rostrocaudal spinal locations may influence the same sympathetic function. Brainstem and spinal neural mechanisms providing excitatory drive to sympathetic neurons that activate homeostatic and metabolic tissues that provide support for movement and exercise and their integration with locomotor neural circuitry are discussed. A unifying conceptual framework for the integrated neural control of locomotor and sympathetic function is presented which may inform future research needed to take full advantage of SCS for improving these spinally mediated autonomic functions.

## Graphical abstract

**Figure:**
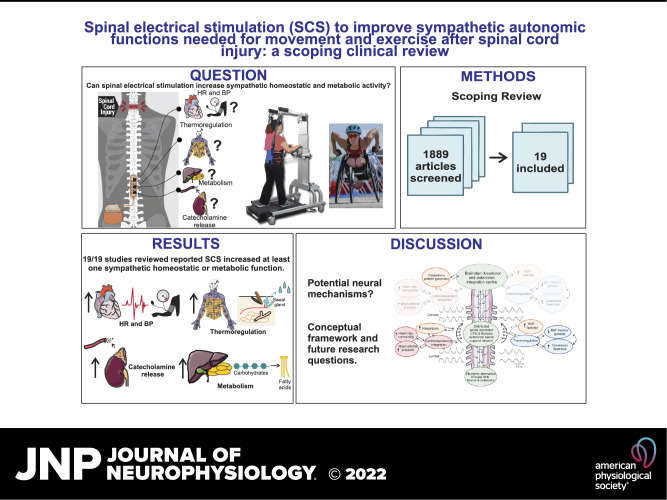

## INTRODUCTION

**Figure 1.**
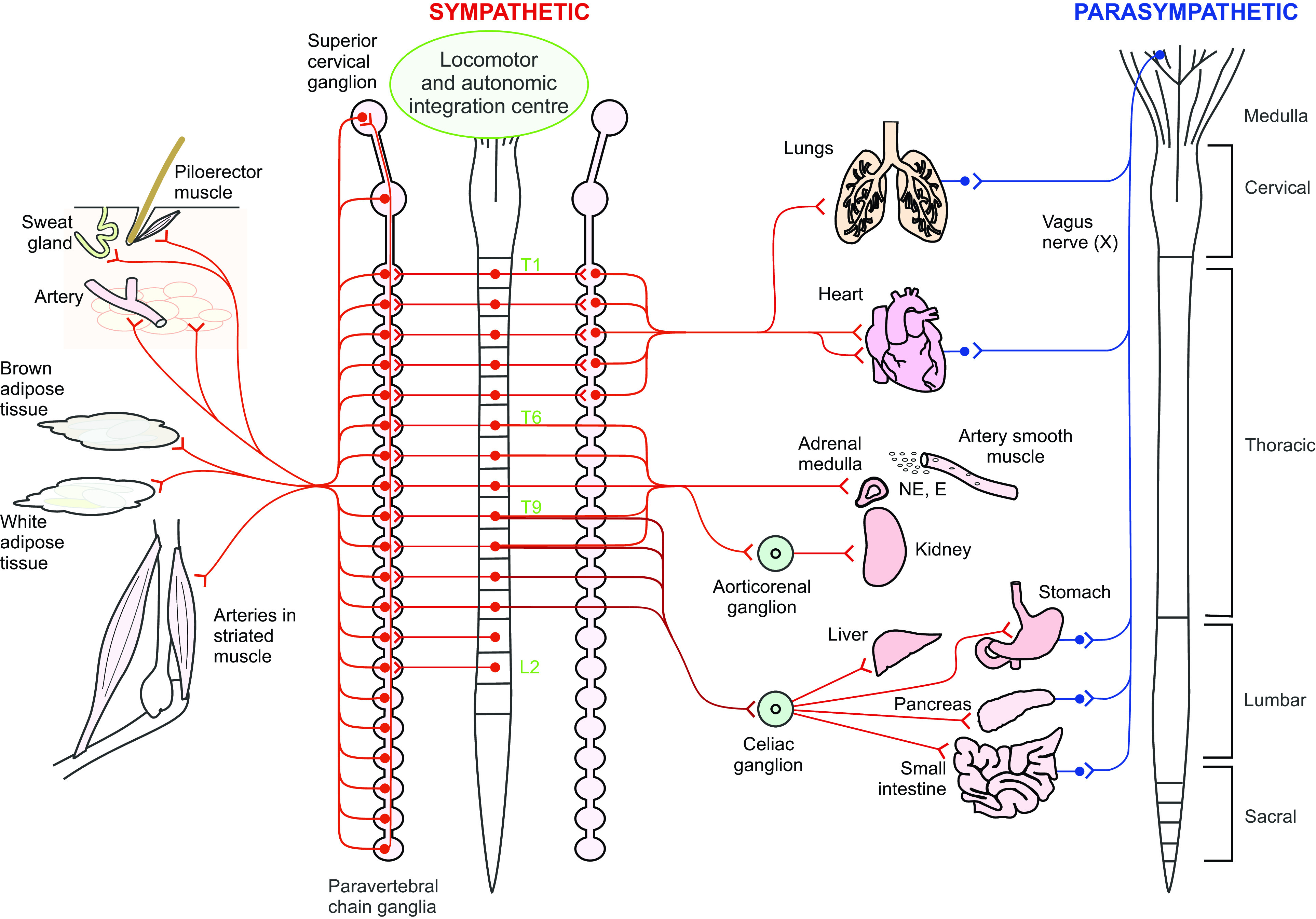
Schematic of central and peripheral localization of preganglionic and postganglionic parasympathetic and sympathetic homeostatic and metabolic autonomic input to key tissues and organs supporting movement and exercise. Spinal levels providing sympathetic input to the lungs, heart, adrenal medulla, sweat glands, white and brown adipose tissue, and smooth muscle of arteries of the splanchnic regions and skeletal muscle tissue are shown in red. Organs that also receive parasympathetic input shown in blue. During movement and exercise parasympathetic input is reduced, limiting digestion, urine production etc., while sympathetic drive to these sympathetic targets is increased. Reprinted with permission from Cowley (8).

Traumatic spinal cord injury (SCI) is a life-altering event for over 40,000 Canadians and 300,000 Americans, and has an estimated global incident rate of 23 per million per year (1, 2). In addition to paralysis and loss of sensation, persons living with SCI face a lifetime of impaired autonomic bodily functions that affect every aspect of daily living. Although we often refer to motoneurons as the “final common path” for executing movement, in essence, the spinal cord itself is the “final common path” for the regulation of virtually every bodily function, and its autonomy and basal level of function is underappreciated when it comes to improving functional outcomes after SCI. Autonomic functions rank very high on the list of research priorities for persons living with SCI, with exercise ranking as important to 96.5% of persons surveyed (3). In relation to this, there is a major class of movement and exercise-related spinally mediated homeostatic and metabolic autonomic functions that are often overlooked when investigating treatments for improving life quality and function after SCI and which contribute to myriad negative health consequences, including obesity, cardiovascular disease, and type II diabetes, which occur earlier and at much higher rates than the general population (4–7). These diseases result not only from lower limb paralysis, but also because persons with cervical level SCI cannot generate appropriate autonomic (i.e., sympathetic) responses during exercise. Such responses normally include increased heart rate (HR), blood pressure (BP), sweating and appropriate metabolic substrate mobilization and use by active muscles (Fig. 1). This constellation of responses is ordinarily evoked by thoracic spinal sympathetic neuronal systems activated by the hypothalamus and mediated by brainstem and spinal autonomic centers via innervation and regulation of pre- and postganglionic fibers to peripheral tissues including the heart, lungs, adrenal glands, fat stores, vasculature, and sweat glands (Fig. 1). Approximately 50% of persons with SCI survive injury at cervical levels (1), after which there is often a complete loss of communication between spinal thoracic autonomic centers and supraspinal regions that mediate acute responses needed to maintain exercise within the cardiovascular, musculoskeletal, endocrine, and exocrine (sweat gland) systems. As a result of these losses, even highly trained Paralympic level athletes with tetraplegia do not produce sufficient exercise responses, with peak HR of ∼≤130 beats/min and peak power outputs, oxygen uptake, and circulating catecholamine levels less than one-third to one-half the values observed in high thoracic (T1–T4 level athletes) (8). From a movement or motor control standpoint, there are no significant differences between low cervical and high thoracic injury (i.e., T4). What differs between injury at C8 versus T4 is the ability to produce a partial sympathetic response during movement and exercise.

Recent progress in clinical SCI research has demonstrated that both epidural and transcutaneous electrical spinal stimulation are powerful therapeutic tools for improving locomotor functions lost after motor complete SCI, even years after injury (9–11). Epidural and transcutaneous stimulation have also been reported to beneficially influence autonomic functions, including bladder, bowel, sexual, and even immune function (12–14). An unanticipated finding of clinical studies aimed at improving locomotor function has been that electrical stimulation of lumbar spinal regions also improved many key metabolic and homeostatic autonomic body functions mediated by spinal thoracic sympathetic neural circuitry. These functions included anecdotal and/or case reports of improved BP regulation during orthostatic challenge, cardiovascular function, temperature regulation, and even exercise performance (15–20).

Focused investigations of movement and exercise-related homeostatic and metabolic autonomic functions have only recently begun to appear in the research literature, and the degree to which spinal stimulation can improve these outcomes is unclear. Given that many of the key organs and tissues mediating whole body homeostatic and metabolic functions needed to maintain movement and exercise receive either only thoracic sympathetic input (i.e., arterial smooth muscle, sweat glands, and adrenal medulla) or a strong direct innervation by thoracic spinal preganglionic neurons (SPNs) activated during exercise (visceral and leg fat stores, the heart, and lungs), it is likely that these sympathetic targets are directly or inadvertently activated by spinal stimulation aimed at increasing either lower or upper limb motor output. Thus, the focus of this systematic scoping review was to summarize the clinical electrical spinal stimulation research literature that reported effect(s) on these autonomic functions. Many of the research laboratories reporting on these outcome measures received training and/or collaborated with the Edgerton laboratory, and these works follow in his career-long interest to understand and reveal the great capacity of the spinal cord for generating locomotor activity and for its plasticity in response to training after SCI.

## METHODS

We used the methodology by Arksey and O’Malley (21) to identify research questions and guide study selection and conformed to Preferred Reporting Items for Systematic Reviews and Meta-Analyses extension for Scoping Reviews (PRISMA-ScR) (22).

### Search Strategy

Literature searches were conducted for English-language journals on July 9, 2020 and repeated in May 2021. The search required the phrases “spinal cord injury” or “SCI” or “tetrapleg*” or “parapleg*,” and “electrical spinal stimulation” or “epidural stimulation” or “transcutaneous stimulation” in the title and/or abstract. Searches were conducted using the Medline, EMBASE, Scopus, CINAHL, and SportDiscus and Cochrane databases, and all restricted to the adult population. The Medline database search required the phrases “spinal cord injury” or “tetraplegi$”or “paraplegi$” and one of the following stimulation-related MeSH terms: “electric stimulation or electric stimulation therapy” or “transcutaneous stimulation or transcutaneous electric nerve stimulation” or “epidural stimulation.” The EMBASE database search required the phrases “spinal cord injury” or “tetraplegi$” and “paraplegi$” in the title and/or abstract, and one of several electrical stimulation-related MeSH terms (spinal cord stimulation, electrical spinal stimulation, transcutaneous stimulation, transcutaneous nerve stimulation, and epidural stimulation). The Scopus database search required the MeSH term “spinal and cord and injury” or “tetraplegi*” and “tetraplegi*” or “quadriplegi*,” and one of several electrical stimulation-related MeSH terms (electrical and spinal and stimulation or epidural or transcutaneous) in the title and/or abstract or both. The CINAHL database search required the MESH term “spinal cord injury” or “SCI” or “paraplegia” or “tetraplegia” or “quadriplegia” or “paraplegic” or “tetraplegic” or “quadriplegic,” and one of several electrical stimulation-related MeSH terms (electrical spinal stimulation or spinal cord stimulation or transcutaneous spinal stimulation or epidural stimulation or epidural spinal stimulation) in the title and/or abstract or both. The SportDiscus database search required the MeSH term “SCI” or “spinal cord injury” or “tetraplegia” or “paraplegia” or “quadriplegia” or “paraplegic” or “quadriplegic,” and one of several electrical stimulation-related MeSH terms (“spinal cord stimulation” or “electrical spinal stimulation” or “transcutaneous spinal stimulation” or “epidural stimulation” or “epidural spinal stimulation”) in the title and/or abstract or both. The Cochrane database search required the phrases “spinal cord stimulation”.

Articles returned from each database were as follows: Medline (247); EMBASE (270); Scopus (267); CINAHL (804); SportDiscus (949); and Cochrane (17). After deduplication 1,889 articles remained.

### Data Extraction

**Figure 2.**
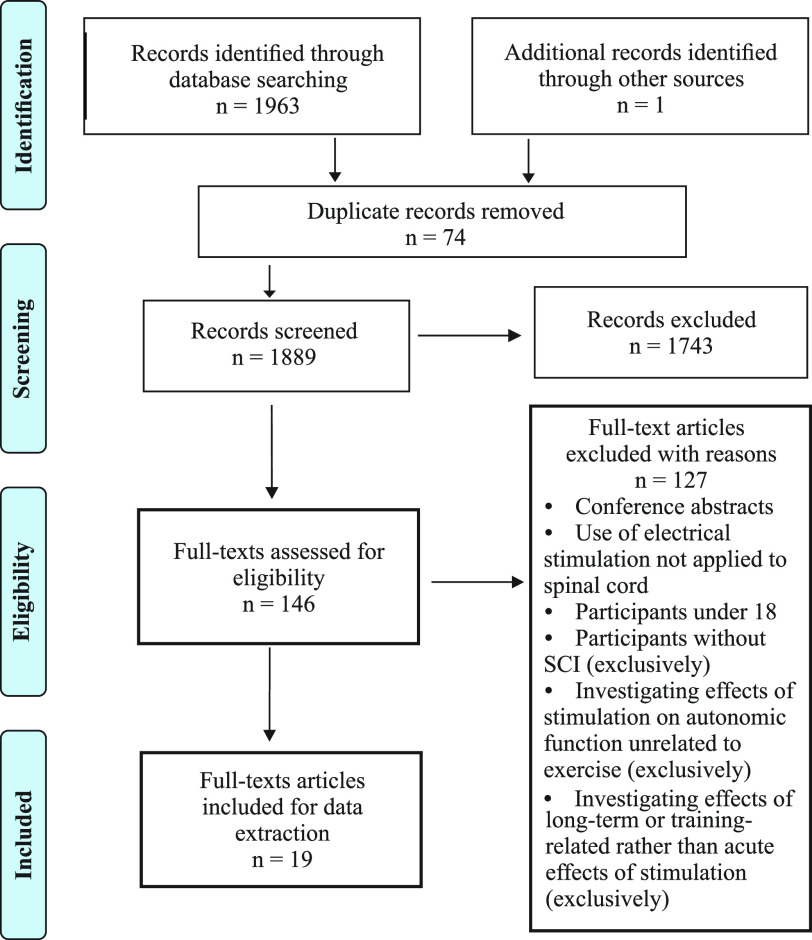
PRISMA diagram showing steps in systematic scoping review, inclusion, and exclusion criteria and numbers of papers at each stage of review. PRISMA, Preferred Reporting Items for Systematic Reviews and Meta-Analyses.

Relevant articles were uploaded to Covidence and duplicates removed. Two researchers working independently reviewed titles and abstracts. A third reviewer settled disagreements on whether the article should be included in the second selection stage (KC). Similarly, full texts of articles were then reviewed independently, and conflicts decided by a third review (KC). A study was included if it involved at least one human participant with an SCI and if any of the following were mentioned in the article: temperature regulation/sweating, blood pressure (BP), heart rate (HR), exercise-testing measures (ratings of perceived, oxygen/energy consumption, circulating catecholamines, or other measures of fatigue). Studies were included if this information was reported as a subjective self-reported outcome, an anecdotal observation, for safety considerations, or if these outcomes were a primary focus of the research study. Studies were excluded if they were conference abstracts; used nonspinal electrical stimulation (i.e., peripheral nerve electrical stimulation); included participants under 18; used only animal data; did not include any participants with SCI; did not examine acute effects of stimulation and only examined long term training adaptations to prolonged use of SCS; or investigated only effects of electrical spinal stimulation on autonomic functions not related to exercise (i.e., bladder, bowel, sexual, or immune function). Participant data extracted included the number of participants, gender, age, time since injury, American Spinal Injury Association impairment scale (AIS) classification, and neurological level of injury. Transcutaneous and epidural stimulation outcome data were summarized separately. Extracted data included author(s), publication date, article title, study design, intervention protocol, electrode placement, electrode size, stimulator, stimulus parameters (frequency, intensity, waveform, and pulse length), treatment length, and exercise-related autonomic outcomes. Results were summarized chronologically and organized by primary focus of investigation (motor function, autonomic function(s), or both). References from selected articles were also reviewed for any relevant articles. Figure 2 provides a PRISMA flow diagram describing the studies identified, excluded and included. Table 1 provides participant characteristics of the 19 studies selected for review.

**Table 1.** Participant characteristics for transcutaneous and epidural stimulation studies focusing on autonomic, motor, or autonomic and motor outcomes

Study [T or E]	Number of Participants	Gender	Age, Yr	Injury Duration, Yr	AIS Classification, *n*	Level of Injury	Study Type
Sachdeva et al. (23) [T1]†	1	1 M	37	3	A	C4	CR
Wu et al. (24) [T2]‡	13	10 M, 3 F	21–65	1–20	B:3, C:5, D:5	C2–C8	CS
Sayenko et al. (25) [T3]‡	15	12 M, 3 F	23–53	2–13	A:11 B:1 C:3	C4–T12	DBCO
Gad et al. (26) [T4]‡	6	5 M, 1 F	20–62	1–21	B:2, C:4	C4:3, C6:2, C8:1	CS
Phillips et al. (20) [T5]†	5	4 M, 1 F	23–32	3+	A:3, B:2	C5–C6, T2	CS
Gad et al. (27) [T6]§	1	1 M	35–40	7	A	T9, L1 (V)	CRCO
Murray and Knikou (28) [T7]‡	1	1 M	27	9	C (UL), B (LL)	C6/C7	CR
Shelyakin et al. (29) [T]‡	25	N R	NR	NR, chronic	NR	NR	CS
Squair et al. (30) [E]†	1	1 M	38	1	A	C5	CR
Darrow et al. (31) [E]†	2	2 F	48, 52	5, 10	A	T4, T8	CS
Nightingale et al. (19) [E]†	1	1 M	33	5	B	C5	CR
Aslan et al. (32) [E]†	7	7 M	26.7 + 4.1	2–3.5	A:4, B:3	C5–T4	PAR
DiMarco et al. (33) [E]§	1	1 M	50	2	NR	C4	CR
Harkema et al. (15) [E]†	4	3 M, 1 F	24–35	3.8–8	A:3, B:1	C4	CS
West et al. (34) [E]†	1	1 M	Early 30s	Chronic, >1	B	C5	CR
Edgerton and Harkema (35) [E]‡	1	1 M	23	>3.5	B	C7–T1	CR
Ganley et al. (36) [E]‡	2	2 M	43; 48	3.5; 8	C	C5–C6, T8	CS
Carhart et al. (37) [E]‡	1	1 M	43	3.5	C	C5–C6	CR
Herman et al. (16) [E]‡	1	1 M	43	3.5	C	C5–C6	CR

**Figure 3.**
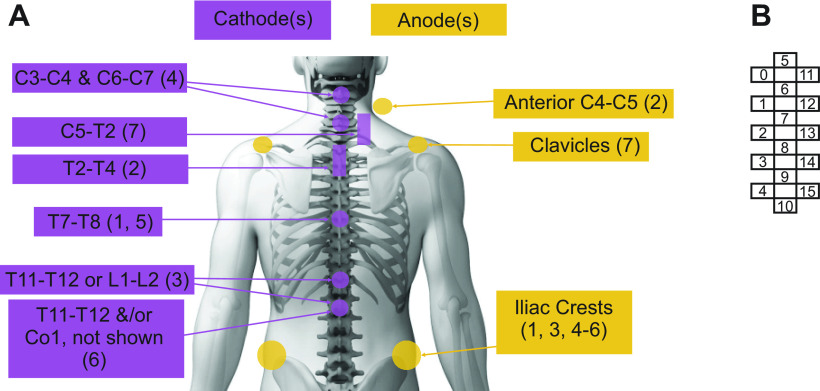
*A*: location of cathodes and anodes for each TSCS study. Numbers in parentheses correspond with study numbers shown in Table 1 and Table 2. *B*: number and position of leads on 5-6-5 paddle electrode used in most ESCS studies. The configurations for cathode and anode lead(s) used in each study and/or participant, if described, are shown in Table 3. ESCS, epidural spinal cord stimulation; TSCS, transcutaneous spinal cord stimulation.

Numbers 1 through 7 correspond to anode and cathode electrode positions as shown on the body image in Fig. 3*A*. All spinal levels are neurological level of injury, unless indicated otherwise with V for vertebral. *n* refers to number of persons with each American Spinal Injury Association impairment scale (AIS) classification when studies include multiple participants. †Autonomic outcome; ‡motor outcome; §autonomic and motor outcome. CR, case report; CRCO, case report, cross over design; CS, case series; DBCO, double blind crossover; E, epidural; LL, lower limbs; NR, not reported; PAR, parallel group design; T, transcutaneous; TSCS, transcutaneous spinal cord stimulation; UL, upper limbs; V, vertebral.

Finally, we performed a search of clinicaltrials.gov to identify any planned or current studies using spinal electrical stimulation in persons living with SCI investigating exercise-related autonomic functions. Search terms included: “spinal cord injuries” and “spinal stimulation,” with results limited to adult participants. This search was first performed on July 22, 2021 and retrieved 311 results. After screening, 17 studies were deemed relevant. This search was repeated on April 28, 2022 and retrieved one additional result. These data were grouped by stimulation method (transcutaneous or epidural) and then by study status (not yet recruiting, active not recruiting, recruiting or completed). Study location, relevant primary research outcomes, anticipated participant numbers, and other study details are provided as an appendix in Table A1.

## RESULTS

Our searches of the above listed databases yielded a total of 1,889 articles (Fig. 2). The titles and abstracts of the 1,815 articles remaining after deduplication were screened for relevance. From this, 146 full text articles were assessed for eligibility. Following the full-text screening, 127 were excluded for reasons noted (Fig. 2) and 19 studies were included for review.

### Characteristics of Articles and Participants

Initial reports on the use of either transcutaneous (T) or epidural (E) electrical spinal cord stimulation (SCS) aimed at improving stepping in persons with SCI appeared in the early 2000s and included anecdotal reports of improvements in sympathetic autonomic functions that support movement, such as increased HR and whole body metabolism (16, 29, 36, 37). Since then, increasing numbers of studies have appeared, but to date, participant numbers are low and consist mainly of case reports (*n* = 10) or case series (*n* = 7; Table 1). Reports regarding homeostatic and metabolic function(s) were mainly anecdotal (11/19), related to safety and tolerability data or outside of the primarily motor outcomes of the study [*n* = 6/8 transcutaneous spinal cord stimulation (TSCS), Table 2; *n* = 5/11 epidural spinal cord stimulation (ESCS), Table 3). The number of participants ranged between 1 and 25 (*n* = 67 participants received TSCS; *n* = 22 received ESCS, although several reports included the same participants, and ages ranged from 20 to 65 (Table 1). Study participants were mainly male (52 M:11 F) and for the 62 described, classified as AIS A (26), AIS B (15), AIS C (15), B/C (1), or AIS D (5). Of note, reports describing systematic or planned examination of the effects of spinal electrical stimulation on autonomic functions that support movement did not appear in the literature until 2018 and represent 2/8 transcutaneous and 6/11 epidural studies (15, 19, 20, 23, 30–32, 34). Stimulation sites for the TSCS studies varied (Fig. 3) as did lead configurations and spinal placement for ESCS studies (described in Table 3).

**Table 2.** Transcutaneous electrical spinal stimulation studies demonstrate stimulation at multiple spinal sites using a variety of stimulation parameters can ameliorate spinal sympathetic dysfunction(s)

Study	Protocol	Electrode Placement, Number, and Size; Stimulation Parameters and Stimulator	Autonomic Outcome(s)
1. Sachdeva et al. (23)	Protocol A (TSCS to prevent AD): *1*) Digital anorectal stimulation (DARS) without TSCS *2*) TSCS then DARS Protocol B (TSCS to interrupt AD): *1*) DARS to trigger AD then *2*) TSCS initiated 30 s after DARS	C: T7–T8 (V, P) 1 × 3 cm round A: L + R iliac crests 2: 5 × 9 cm Freq: 30 Hz Width: 2 ms Shape: rectangular (rect), biphasic Amp: 20–30 mA Digitimer DS5	**TSCS both prevented and reduced DARS-induced increase in SBP** Protocol A Prevention: *1*) without TSCS, DARS ↑ SBP by 29 ± 5 mmHg: AD is ↑ SBP > 20 mmHg *2*) with TSCS, DARS ↑ SBP by 5 ± 3 mmHg Thus DARS-induced ΔSBP was 82% less when preceded by TSCS* Protocol B Treatment: *1*) without TSCS, DARS ↑ SBP by 18 ± 1 mmHg *2*) after TSCS, SBP ↓ by 9 ± 4 mmHg Thus, TSCS reduced DARS-induced rise in SBP by ∼50%, when applied after DARS *TSCS also reduced DARS-induced reflex pelvic floor EMG activity
2. Wu et al. (24)	TSCS was delivered intermittently while seated, at rest, for the duration of the motor study (duration not stated). Study examined the effects of single and paired pulses of TSCS on the latency and amplitude of motor responses in muscles of the arm and hand.	C: T2–T4 V, P 5 x 10 cm placed longitudinally A: C4–C5 V, A 5 × 10 cm placed horizontally ∼2–3 cm superior to sternal notch Grounds: L + R clavicles, 2: 5 × 10 cm Freq: 0.2 Hz Width: 2 ms Shape: rect, biphasic Amp: 4.4–102 mA, 100—175% of resting motor threshold (RMT) of the abductor pollicis brevis (APB) Digitimer DS7A or DS8R	**Safety and Tolerability Data** Changes to resting BP ↑ MAP > 20% over baseline for at least 15 min (*n* = 7/13 with SCI and in 0/14 able-bodied (AB) controls)* ↓ MAP by 20% or more from baseline for at least 15 min (*n* = 2/13 SCI and 0/14 AB) Changes to resting HR ↑ HR by 20% or more from baseline for at least 15 min (*n* = 1/13 SCI and 0/14 AB) ↓ HR by 20% or more from baseline for at least 15 min (*n* = 7/13 SCI and 1/14 AB) *Authors reported these increases to be like those seen during peripheral nerve stimulation alone. These increases appeared solely or predominantly in those with cervical SCI and not in either AB controls or participants with ALS (*n* = 4).
3. Sayenko et al. (25)	All participants received TSCS, no stimulation, and “sham” TSCS during assisted standing in a custom frame to assess ability to stand. 1 testing day (2 h, *n* = 15/15), and/or 12 training sessions (2 hours, *n* = 6/15)	C: T11–T12 or L1–L2 V, P, 3.2 cm round A: L + R iliac crests 2: 7.5 × 13 cm Freq: 15 Hz* Width: 1 ms, filled with 10 kHz carrier wave Shape: rect, monophasic Amp: <150 mA Custom built constant current stimulator *selected as most effective for standing, [tested 0.2–30 Hz]	**Safety, Tolerability, and Anecdotal Data** ↑ sweating below lesion during first session (*n* = 3/15) Authors reported that if symptoms of OH occurred, they were readily mitigated by adjusting stimulation intensities No bouts of AD were observed ↑ SBP by more than 60 mm Hg during first session at a very low stimulation intensity (10 mA), with no further incidents following first session (*n* = 1/15)
4. Gad et al. (26)	4-wk (2×/wk) hand grip strength program with TSCS. TSCS delivered during 18 MVC attempts in middle of each 1–2 h training session	C: *1*) C3–C4 and *2*) C6–C7 V, P 2: 2 cm round A: L + R iliac crests 2: 5 × 10 cm Freq: 30 Hz filled with 10 kHz carrier wave Width: 1 ms Shape: rect, biphasic (for AIS C) or monophasic (for AIS B) Amp: 10–250 mA NeuroRecovery Technologies multi-channel stimulator	**Anecdotal Data** ↑ sweating ability generally or below level of lesion (*n* = 2/6)
5. Phillips et al. (20)	Protocol to elicit orthostatic hypotension (OH) and then assess if TSCS can treat OH-related symptoms: *1*) 25 min rest, supine, then; *2*) Induce orthostatic challenge (OC: tilt table) and when systolic BP ↓ by 20 mmHg then; *3*) TSCS	C: T7/T8 V, P 3 cm round A: L + R iliac crests 2 × 5 × 9 cm Freq: 30 Hz Width: 1 ms Shape: rect, monophasic Amp: 10–70 mA until BP normalized (>1 min) Stimulator NR	**TSCS normalized OH-induced decreases in BP and cerebral blood flow** TCSC increased SBP, DBP, MAP, and middle and posterior cerebral artery blood flow to baseline levels (after OC reduced these measures) OH symptoms (e.g., dizziness) were reduced with TSCS HR became elevated, CO and SV were reduced with OH and were not changed with TSCS during OC *Authors noted that leg muscle EMGs were recorded to confirm no activation and therefore no TSCS-induced pressor-related effects on BP
6. Gad et al. (27)	*1*) Participant walked using EKSO alone for 4 wk, then; *2*) 1 wk of EKSO + TSCS, then; *3*) 1 wk of EKSO + monoamine agonist buspirone (10 mg, 2/day), then; *4*) 1 wk of EKSO + TSCS + buspirone. Training = 1 h/day, 5 days/wk.	C: T11/T12 and/or Co1 V, P, 2.5 cm round A: L + R iliac crests 2: 5 × 10.2 cm Freq: 30 Hz over T1/12 5 Hz over Co1 Width: NR* Shape: NR* Amp: determined by efficacy and comfort* Stimulator NR*	**Anecdotal HR, BP, and self-reported sweating outcomes** HR during stepping with EKSO alone increased minimally with EKSO + TSCS (by ∼10 beats/min, from ∼70 to ∼80 beats/min) and by ∼10 beats/min with EKSO + buspirone (to ∼90 beats/min) but ↑ substantively during stepping with TSCS + buspirone (to ∼135 beats/min) BP during stepping generally similar throughout protocol (∼ 140/95 mmHg EKSO alone; ∼ 150/90 EKSO + TSCS; 152/92 EKSO + TSCS + buspirone) ↑ self-reported sweating score increased from 1/5 (EKSO alone) to 3/5 (TSCS alone) to 5/5 (TSCS + buspirone) *based on reference to 38, assume stimulation is with 10 kHz carrier wave; Width: 0.3—1 ms; Shape: rect, biphasic; Amp: 30—200 mA
7. Murray and Knikou (28)	*1*) Baseline TMS-elicited motor evoked potentials recorded in extensor and flexor carpi radialis muscles; single and dual pulse (inter-pulse interval ranged: 1–30 ms) stimulus paradigms, then; *2*) Participant received ∼55 min of TSCS while supine for 14 sessions (over a 3 wk period); to avoid exhaustion and facilitate spontaneous neuron depolarization, stim delivered in 10 min blocks, then; *3*) TMS protocol repeated after treatment period	C: C5–T2 V, P 10.2 × 5.1 cm, placed vertically A: L + R clavicles 10.2 × 5.1 cm Freq: 0.2 Hz Width: 1 ms Shape: rect, monophasic Amp: intensities ranged from 5–68, mean 42 mA. Intent was to use intensities ranging from below motor threshold to intensities that evoked bilateral muscle contractions Digitimer DS7A triggered by CED Spike 2 scripts	**Anecdotal self-reported sweating outcome** During the intervention (Protocol step 2) participant reported he had started to sweat in the upper back and armpits, a response that had stopped after the injury
Shelyakin et al. (29)	*1*) Electrode placement was individualized, NR *2*) Treatment consisted of 20 sessions (20–50 min each) of direct current stimulation *3*) Motor outcomes were different for each of the 25 patients with chronic SCI or tuberculous spondylitis	C and A: Placed along the vertebral column, “positions and distance between electrodes were changed depending on the clinical effect obtained and the nature of changes in electrophysiological measures” Agar electrodes, 600 mm^2^ Direct current stim Amp: <10 mA	**Anecdotal HR Outcomes** ↑ HR from 95–100 (baseline) to up to 120 beats/min during direct current stimulation treatment sessions, authors proposed this effect “is probably due to the direct action of the direct current on ganglia of the sympathetic nervous system located along the spinal cord.”

A, anode; ant, anterior; AD, autonomic dysreflexia; C, cathode; CO, cardiac output; EMG, electromyogram; EKSO, exoskeleton; HR, heart rate; MAP, mean arterial pressure; NR, not reported; OC, orthostatic challenge; OH, orthostatic hypotension; P, posterior; PT, physical therapy; S, spinal; SBP, systolic blood pressure; SV, stroke volume; TMS, transcranial magnetic stimulation; TSCS, transcutaneous spinal cord stimulation; V, vertebral.

**Table 3.** Epidural electrical spinal stimulation studies demonstrate stimulation within lower-thoracolumbar and sacral spinal regions can ameliorate multiple spinal sympathetic dysfunction(s)

Study	Protocol	Electrode Placement and Configuration; Stimulation Parameters and Stimulator	Autonomic Outcome(s)
Squair et al. (30) 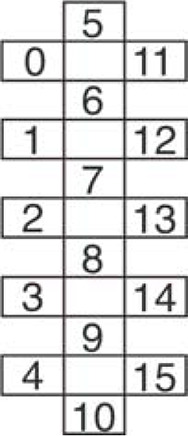	*1*) Simulation parameters that elicited a rise in resting systolic BP compared. Selected parameters eliciting ↑ of ∼40 mmHg for use in orthostatic challenge (OC) in step 2. Then: *2*) OC with and without ESCS	T10–L1 D, S Electrode: 3-Col (5-6-5 lead) 16-contact array Cathode (C): 0–1, 11–12 Anode (A): 5–6 Freq: 120 Hz Amp: 0–7.5 mV Width: 450 µs Intensity determined by degree of reduction in BP during orthostatic hypotension (OH) Stimulator: Medtronic RestoreAdvanced SureScan neurostimulator	**ESCS normalized OH-induced decreases in BP and could increase resting systolic BP by ∼40 mmHg ** *1*) Testing configurations for ↑ systolic BP at rest: *i*) ESCS ↑ resting systolic BP by ∼40 mmHg: C: 0–1, 11–12 A: 5–6 *ii*) ESCS ↑ resting systolic BP by ∼13 mmHg: C: 2, 13 A: 8 *iii*) ESCS ↑ resting systolic BP by ∼5 mmHg: C: 3–4, 14–15 A: 9–10 *iv*) ESCS ↑ resting systolic BP by ∼0 mmHg: C: 5–6 A: 0–1, 11–12 Configuration *i*) selected as most effective for f↑ BP, and used during OC. 2. ESCS could repeatedly interrupt episodes of OH during OC. Other: Reported serum noradrenaline increased from ∼0.2 to 0.6 nmol/L after ESCS
Darrow et al. (31) 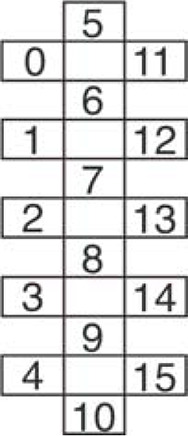	*1*) Implantation of electrode used intraoperative monitoring of leg muscle EMG and was secured in place where maximal and symmetric EMG observed with lowest stimulation current. *2*) BP and HR were monitored during orthostatic challenge (OC: 70 degrees tilt-table test). SCS applied once orthostatic hypotension (OH) observed with and without SCS.	L1–S2 D, S Electrode: 3-Col (5-6-5 lead) 16-contact array Freq: 50 Hz Amp: 5 mA Width: 350 µs Participant (P)1 (no OI): C: 0, 6, 11 A: 4, 10, 15 P2 (OI): C: 0, 5, 11 A: 4, 9, 15 Stimulator: Primary cell internal pulse generator (Tripole and Proclaim Elite).	**SCS normalized cardiovascular responses during OC in participant with OI and did not alter responses in person without OI** P1 (no OI): no change in BP or HR during OH challenge, and not altered by introduction of SCS during tilt-table test P2 (OI): SBP ↓ by mean ∼30–40 mmHg with OC, SCS interrupted OI and ↑ SBP by ∼ mean 30–40 mmHg to baseline values OC ↓ cerebral blood flow (CBF) from ∼6 cm/s to ∼5 cm/s and SCS ↑ CBF by ∼ 1 cm/s to baseline values Cognitive function outcome measures improved after SCS during OC (Digit span, Stroop and Verbal fluency tests)
Nightingale et al. (19) 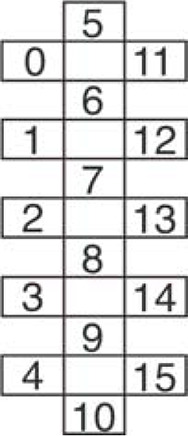	Six progressive arm crank ergometry tests to exhaustion (V̇o_2peak_), separated by 12 days. Each of 3 conditions [*1*) no ESCS, *2*) abdominal (AB) ESCS or *3*) cardiovascular (CV) ESCS repeated twice]. Order of testing randomly assigned with assessors blinded as to trial allocation.	T11–L1 D, V Electrode: 3-Col (5-6-5 lead) 16-contact array AB parameters: Freq: 40 Hz Amp: 3.5–6.0 V Width: 420 µs C: 1, 6, 12 A: 0, 5, 11 CV parameters: Freq: 35 Hz Amp: 3.5–6.0 V Width: 300 µs C: 1, 4, 6–10, 12, 15 A: 0, 2, 3, 5, 11, 13, 14 * same participant as in 34	**Exercise related autonomic outcomes** Both AB and CV ESCS: ↑ V̇o_2peak_ (absolute and relative; 15%–26%) ↑ Peak ventilation (from 33 to ∼50 L/min with SCS) ↑ Peak oxygen pulse (8%–13 % for low intensity SCS, 21% for high intensity SCS) ↓ RPE at sub-peak power output of 60 W (from 18/20 to 14–15/20) In addition, CV ESCS: ↑ MAP by 14 mm Hg at rest (cardio program) regardless of intensity
Aslan et al. (32) 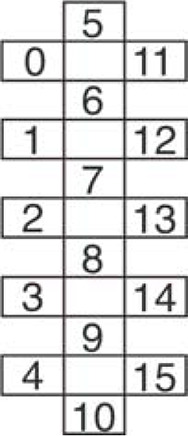	*1*) Before electrode implantation, orthostatic intolerance (OI) was determined as follows. While supine, blood was drawn for baseline serum catecholamine concentrations, then repeated at 3′ and 10′ of sitting orthostatic challenge (OC). BP and HR were monitored continuously throughout testing. Participants were separated into two groups: *1*) OI, if they demonstrated resting hypotension, OI to sitting challenge and low levels of circulating catecholamines or *2*) no OI, if the above were not observed. *2*) Monitored resting supine BP and leg EMG responses to increasing ESCS amplitude at each of rostral and caudal ESCS configurations. *3*) Monitored BP and HR during ESCS-induced standing before and after transition from sit to stance. Stimulation parameters and electrode configuration individually determined but selected specifically to elicit motor activity with cathode in caudal ESCS configuration.	L1–S1 D, S (T11–L1 V) Electrode: 3-Col (5-6-5 lead) 16-contact array. C, A, and Freq Individualized*. Grp 1 *B23: 30 Hz, C = 7, 10, 13, A = 2, 4, 15 *B13: 15 Hz, C = 4, 10, 15, A = 9 *B07: 15 Hz, C = 4, 10, 15, A = 3, 9, 14 Grp 2 *A60: 25 Hz, C = 4, 10, 14, A = 3, 12 *A59: 25 Hz, C = 4, 10, A= 6, 12 *A53: 35 Hz, C = 4, 10, 15, A = 3, 8, 14 *A45: 25 Hz, C = 4, 10, 14, A = 3 Stimulator: Medtronic 5-6-5 Specify with RestoreADVANCED pulse generator	**ESCS normalized cardiovascular responses during OC in group with OI and did not alter responses in group without OI** Group 1 (OI: all AIS B, C5–T2 SCI) SCS ↑ BP while supine and amplitude of BP response could be altered by changing cathode leads from rostral to caudal and vice versa SCS ↑ BP to within normal ranges during standing and prevented decline in BP seen with transition from sitting to standing without SCS Group 2 (no OI: all AIS A, T4 SCI) No change in BP with SCS while supine, regardless of rostral or caudal cathode configuration BP maintained during transition from sit to stand, response not altered by SCS
DiMarco et al. (33)	*1*) Electrode implantation to restore cough function. *2*) Participant instructed to apply stimulation every 30 s for 5–10 min, 2 or 3 times/day, in the home to help with expiratory airflow. *3*) Participant was able to cough when using SCS. Peak expiratory airflow rate and maximum pressure increased and plateaued over 6–8 wk. Was followed for > 1.5 yr.	T9–T11 D, S, just lateral to midline on L and R Freq: 50 Hz Amp: 40 V Width: 0.2 ms Waveform: biphasic Electrode: 4 lead, bilateral Implanted receiver (Finetech Medical Ltd) activated by an external transmitter, controlled by a portable hand-held stimulator.	**Anecdotal Data** BP ↑ to 175 mmHg in first ESCS session HR ↓ to 55 beats/min in first ESCS session Cardiovascular responses gradually abated and then disappeared with repeated use over a 9-wk period.
Harkema et al. (15) 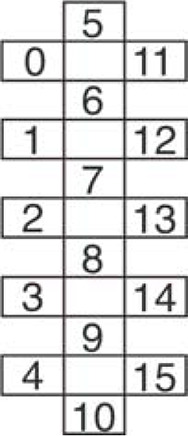	*1*) Stimulation parameters and electrode configuration individually determined over 2–3 initial 2-h sessions while at seated rest with outcome goal of 105–120 mmHg SBP, while minimizing EMG in multiple leg muscles. *2*) Repeat testing of “effective” stimulation leads/parameters on BP, HR at rest over 5 subsequent 2-h sessions.	L1–S1 D, S (T11–L1 V) Freq: 30–65 Hz Amp: 3–7 V; selected based on sys BP of 105—120 mmHg while < motor Thr Width: 450 µs Electrode: 3-Col (5-6-5 lead) 16-contact array. C, A and Freq Individualized*. Stimulator: Medtronic 5-6-5 Specify with RestoreADVANCED pulse generator	**ESCS normalized resting BP in persons with cSCI and OI** induced sustained ↑ MAP (Δ 10–30 mmHg), reproducible over 5 sessions induced sustained normalization of SBP and DBP with ↑ of ∼10–44 mmHg HR remained similar in ¾ and decreased in ¼ All showed ↑ HR from baseline upon SCS cessation Anecdotally, participants reported increased alertness, ability to project their voice, capacity to breathe, and improved sense of well-being with SCS. *A41: ∼30 Hz, C: 0–1, 11–12, A: 5-8 *A68: 50 Hz, C: 0–3, A: 4, 10, 15 *B21: 60 Hz, C: 0–2, 11–14 *A80: ∼65 Hz, C, A: multiple configurations
West et al. (34) 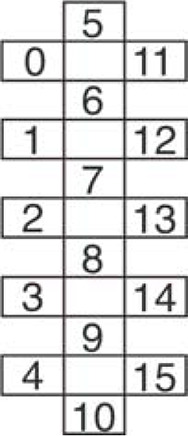	*1*) Using epidural stimulator implanted to improve lower limb stepping function, stimulation parameters, and electrode configuration to increase BP while at seated rest was determined with initial testing over a 2-wk period. Outcome goal of 105–120 mmHg SBP, while minimizing EMG in multiple leg muscles. *2*) 3 testing days consisted of supine and head up tilt manipulation with and without SCS while monitoring beat by beat BP, cardiac function with transthoracic echocardiography, cerebral blood flow with transcranial Doppler, and lower limb EMG.	T11–L1 D, V Electrode: 3-Col (5-6-5 lead) 16-contact array C: 1, 4, 6-10, 12, 15 A: 0, 2-3, 5, 11, 13-14 Freq: 35 Hz Amp: 3.5 V Width: 300 ms Stimulator: Medtronic 5-6-5 Specify with RestoreADVANCED pulse generator	**ESCS normalized cardiovascular responses during orthostatic challenge** ESCS: maintained SBP during head up tilt OH challenge, but was ↓ by 20—30 mm Hg without SCS mitigated ↑ in HR during head up tilt OH challenge (↑ ∼10 beats/min with SCS vs. ↑ ∼40 beats/min without SCS) mitigated ↓ in end diastolic volume during head up tilt OH challenge (↓ ∼20 mL with SCS vs. ↓ ∼50 mL without SCS) mitigated ↓ in cardiac output during head up tilt OH challenge (↓ ∼ 0.3 L/min with SCS vs. ↓ ∼1 L/min without SCS) maintained middle cerebral artery velocity during head up tilt OH challenge, but was ↓ by 8–12 cm/s without SCS
Edgerton and Harkema (35)	Review and commentary of >18-mo observations using epidural stimulation in motor complete T1 participant with SCI.	L1–S1 D, S (T10–T12 V) Electrode: 16-leads Freq: NR Amp: NR Width: NR Stimulator: NR	**Exercise related autonomic outcomes** Normalized BP regulation Improved temperature regultion Non-exercise autonomic outcome included voluntary control of bladder, even without stimulation.
Ganley et al. (36)	Protocol as in Carhart (37) and Herman (16) and includes same participant (P1) + 1 additional participant 2 (P2) had T8 SCI and received FES during PBWT before receiving epidural implant.	T10–T12 D, V, but for P2, leads were placed slightly more lateral due to scar tissue. Freq: 20–60 Hz Amp: Midway between sensory and motor threshold (Thr) for P1 and at motor Thr for P2. Width: 800 µs Stimulator: as in Carhart and Herman	**Exercise related autonomic outcomes** Overground walking with ESCS at each time point tested showed immediate changes for P1 as described in Carhart and Herman. P2 showed similar but less robust effects on speed and endurance as P1. In addition, P2 showed: ↓ O_2_ consumption (∼2,700–1,500 mL/kg/km, to 0.55×) ↓ net carbohydrate oxidation rate (3.8 to 1.2 kcal/min, to 0.32×) ↑ FA oxidation (from 1 to 2.5 kcal/min, to 2.5×) ↓ RER (from 0.91 to 0.78)
Carhart et al. (37)	*1*) Treadmill: partial weight bearing therapy (PWBT), < 2 h/day, 5 times/wk until plateau (from 0.45 m/s, 40% static BW to 0.65 m/s < 20% BW) *2*) Treadmill-PWBT with/without ESCS *3*) Overground training with/without ESCS	Lumbar enlargement, D, S (T10–T12 V) Electrode: 4 cylindrical contacts (6 mm × 1.2 mm), 2 leads arranged in parallel, 1 mm lateral to midline, quadripolar stimulation configuration Freq: 40–60 Hz Amp: Midpoint between sensory and motor Thr Width: 800 µs Waveform: Continuous, charge-balanced monophasic rectangular pulse followed by low amplitude-long duration opposite polarity rectangular pulse.* Stimulator: Medtronic fitted with dual Pisces-Quadplus Model 3888 electrode leads, X-TREL 3470 receiver, X-TREL transmitter (M 3425) and external antenna (M 3440). Transmitter powers implanted receiver via transcutaneous radio frequency telemetry. Pulse generator used in SingleStim mode.	**Exercise related autonomic outcomes** Overground walking with ESCS at each time point tested showed immediate: ↑ in walking speed (2×) ↓ RPE from ∼8/10 to ∼3/10 ↑ walking duration (1.5–2×) * this paper describes testing multiple stimulation parameters and lead configurations (bipolar and tripolar), (0.1–7.0 V), 240–900 µs) and frequencies (10–100 Hz). For each width and frequency, amplitude at midpoint between sensory, and motor threshold produced greatest improvements in motor performance at frequencies between 40 and 60 Hz and widths > 500 µs.
Herman et al. (16)	*1*) Established plateau in gait performance (90% BW, 2.0 mph) using progressive training with partial weight bearing therapy (PWBT) on a treadmill. *2*) Implant epidural stimulator, after healing, re-implement PBWT with ESCS. *3*) Compare mean speed, stepping symmetry, RPE, and whole body metabolic activity with and without ESCS during stepping. Motor: with ESCS smoother stepping pattern at higher treadmill speeds and self-supported body weight, less spasticity.	T11–T12 V 4 contacts, placed 1–2 mm off dorsal midline with span of contacts covering 15 mm to span the entire upper lumbar enlargement. Freq: 20–60 Hz (observed similar responses over this range of frequencies) Amp: > Sens Th, < Mot Thr Width: 800 µs lower durations less effective Stimulator: Medtronic fitted with a pair of Pisces-Quadplus electrodes (with X-TREL stimulation system, Medtronics) inserted into the dorsal epidural space.	**Exercise related autonomic outcomes** Overground walking with ESCS: immediate improvement with increased endurance and speed ↓ RPE from 8/10 to 2/10 ↓ O_2_ consumption to 0.64× for same distance ↑ FA oxidation 8× for same distance ↓ O_2_ consumption to 0.73× for same duration ↑ FA oxidation 5.9× for same duration Thus increased whole body FA oxidation vs. glycolysis for similar or higher intensity stepping

Studies focus on autonomic, motor, or autonomic and motor outcomes. The configuration of lead(s) selected as cathode (C) and anode (A) in each study and/or for each participant are included in columns 3 or 4, if provided. Ab, abdominal; BP, blood pressure; BW, body weight; cMAP, cerebral mean arterial pressure; Co, coccygeal; CO, cardiac output; D, dorsal; ESCS, epidural spinal cord stimulation; FA, fatty acid; FM, fat mass; HI, high intensity; MAP, mean arterial pressure; mCBFv, mean cerebral blood flow velocity; NR, not reported; OC, orthostatic challenge; OH, orthostatic hypotension; OI, orthostatic intolerance; PWBT, partial weight-bearing training; RER, respiratory exchange ratio; RPE, rating of perceived exertion; S, spinal; SCS, spinal cord stimulation; SV, stroke volume; thr, threshold.

### SCS Modulates HR and Whole Body BP, Temperature, and Metabolism Regulation

Reports of improved autonomic functions supporting movement include temperature regulation or reappearance of ability to sweat (25, 26, 27, 28, 35), increases in HR at rest (24, 29, 32), increased speed of movement and/or reduced sense of effort for the same intensity of movement or exercise (16, 19, 36, 37), alterations in whole body metabolism such that carbohydrate metabolic substrate use was reduced and fatty acid (FA) oxidation increased for the same or higher speeds of movement (16, 36, 37) and normalized BP regulation at rest or while standing or stepping (35). With the exception of one study describing a cardiovascular stimulation paradigm eliciting increased resting HR of approximately 14 beats/min (19), most changes in HR were either transient or highly variable (15, 24, 20, 24, 27, 29, 33, 34). Those with injury above T1 and severely limited peak HR would benefit from research determining how and whether it is possible to safely and reliably increase HR during movement and exercise but will not be discussed in great detail in this study.

### SCS at a Variety of Rostrocaudal SC Sites and Parameters Normalized BP Regulation

For those with SCI above ∼T6, regulation of BP during rest and activity is often impaired, with some demonstrating such severe orthostatic intolerance (OI) that rising from supine to sitting can result in BP reductions sufficient to cause loss of consciousness. Each of the five studies investigating either ESCS (15, 30–32) or TSCS (20) demonstrated that it was possible to increase BP during orthostatic challenge (OC). The types of OCs used included being manually raised from supine to sitting, standing up from sitting, or receiving a head-up tilt test while secured to a tilt-table. In the head-up tilt test, the participant is passively raised from horizontal to 75^°^ to 90^°^ (as tolerated) while continuously monitoring BP. Modulation of BP by SCS occurred only in those with impaired BP regulation with no effect on those without OI. Participants were classified as having OI by demonstrating resting hypotension and OI. Fifteen out of 20 persons in these studies displayed OI, were either cervical or high thoracic level injury, and all responded to SCS with increases in BP of 10–40 mmHg (Tables 1, 2, and 3). After stopping stimulation, BP returned to low baseline values. The five participants that did not display OI had lower-level SCI (T4 or below) and did not show any BP changes in response to SCS. Effective epidural stimulator locations included spinal T10–L1 (*n* = 1), L1–S1 (*n* = 8), L1–S2 (*n* = 1), and transcutaneous stimulation at vertebral T7/T8 (*n* = 5). Specific epidural lead configurations were mainly individualized and are described in Table 3. Stimulation parameters also varied, from 1 ms pulse width and 30 Hz frequency for TSCS to ESCS parameters ranging from 15 to 120 Hz with pulse widths of 350–450 µs, and one reporting use of 300 ms. Amplitudes were increased until effects on BP were observed. Stimulation parameters and intensity were often targeted such that BP was improved by the greatest amount while limiting motoneuron activation as evidenced by minimal leg muscle EMG discharge (Tables 2 and 3). Thus, a variety of stimulation parameters, lead configurations, and rostrocaudal electrode locations modified BP responses in persons with OI caused by cervical or high-thoracic level SCI.

Conversely, TSCS reduced BP in response to induced episodes of autonomic dysreflexia (AD) in one case study (23). The same site and similar stimulation parameters as used above to increase BP were used in this case to either prevent or interrupt the rise in BP seen during episodes of AD induced by nociceptor stimulation [TSCS at T7–T8, frequency = 30 Hz, similar pulse width of 2 vs. 1 ms].

### SCS at a Variety of Rostrocaudal SC Sites and Parameters Improved Temperature Regulation

Several studies anecdotally reported improvement in temperature regulation or reappearance of sweating with stimulation at cervical or high-thoracic (26, 28), low-thoracic or lumbar (25), and/or lumbosacral (27, 35) levels (Tables 2 and 3). We did not find any studies systematically examining whether SCS could induce sweating or alter temperature regulation capacity in persons with impaired sudomotor function.

### ESCS Alters Whole Body Metabolism, Ratings of Perceived Exertion, and Peak Exercise Performance

Four studies reported alterations in whole body metabolism, steady state, or peak motor or exercise performance (16, 19, 36, 37). To date only one study, to our knowledge, examined peak exercise performance, with lumbar ESCS repeatedly increasing peak oxygen uptake (V̇o_2peak_) during a progressive arm ergometer test to exhaustion by approximately ≥ 20%, which was associated with increases in peak ventilation and oxygen pulse (an indirect measure of stroke volume) in a person with C5 (AIS B) level tetraplegia. In this case study, two different lead configurations were tested and both increased V̇o_2peak_ by similar levels. One configuration also induced an increase in resting BP of ∼14 mmHg. Ratings of perceived exertion (RPE) for the same intensity of exercise were reduced in trials with ESCS (from 18/20 without to 14–15/20 with SCS). In terms of whole body metabolism, acute application of lumbar ESCS during overground stepping, with partial body weight support, in two persons with motor incomplete cervical or midthoracic SCI caused a reduction in the proportion of carbohydrate oxidation and an increase in the proportion of fatty acid (FA) oxidation. This change corresponded with large decreases in RPE (from ∼8/10 without to 3/10 with ESCS) and increases in endurance (1.5 to 2 times with ESCS vs. without) and speed (2 times with ESCS vs. without). When compared for the same distance or duration of stepping, oxygen/energy consumption was reduced when ESCS was applied (0.64 times with ESCS vs. without) (16, 36, 37).

### Ongoing Studies on Clinicaltrials.gov Indicate Increasing Interest in SCS to Improve Exercise-Related Spinal Autonomic Functions

In addition to the published literature described in the RESULTS, we identified 18 SCS trials currently listed on clinicaltrials.gov, suggesting there is growing recognition of the possibilities for SCS to modulate many key movement and exercise-related autonomic functions. Most (11/18) include BP and/or HR regulation (Table A1), whereas 9/18 focus on or include at least one of thermoregulation, oxygen consumption, catecholamine levels, and sympathetic nerve activity as an outcome measure. Estimated participant recruitment numbers range from 2 to 100 (14/18 are recruiting ≥10 participants), suggesting an increasing recent interest in more systematic investigation regarding SCS to alter these spinal sympathetically mediated functions.

## DISCUSSION

This scoping review identified clinical research studies demonstrating potential for electrical stimulation of the spinal cord to facilitate thoracic spinal autonomic homeostatic and metabolic functions that support movement and exercise. Specifically, lumbar spinal electrical stimulation normalized BP regulation in persons with impaired cardiovascular function due to high thoracic or cervical SCI, facilitated temperature regulation in persons with previously dormant sweating function and altered whole body metabolic substrate use, which was associated with reduced perceived exertion during lower limb movement and upper limb exercise. The ability of electrical stimulation of the lumbosacral SC to improve or elicit stepping, stance and even voluntary movement of the lower limbs in persons with motor complete SCI is now well established and has been reviewed elsewhere (9, 10, 38, 40–44). The potential for SCS to also improve cough, sensation, and other autonomic functions such as bladder, bowel, and immune function has been reported or recently reviewed (12–14, 33, 45–47). Here, we review and discuss underlying spinal neural mechanisms and pathways regulating sympathetic metabolic and homeostatic organs and tissues for cardiovascular homeostasis, cooling temperature regulation and delivery of energy substrates to active skeletal muscle, and their integration with locomotor neural circuitry.

Given the wide range of functions, the spinal cord is responsible for mediating or regulating, it is not surprising that SCS has been reported to influence so many body tissues and organs. Neural mechanisms underlying improvements in the homeostatic and metabolic autonomic functions reviewed are unknown, but there are parallels between electrical activation of thoracic sympathetic neural circuitry and activation of motor circuitry. It is likely that similar neural structures are activated, and in similar recruitment order, in terms of afferents, efferents, and deeper spinal structures. In addition to direct segmental effects elicited through direct activation of sympathetic afferents or preganglionic neurons, there would also be activation of sympathetic circuitry elicited secondary to stimulation of somatic afferents. Finally, there may be activation of SPNs mediated in concert with activation of locomotor-related neurons within central locomotor pattern generator (CPG) circuitry as part of an integrated spinal locomotor-sympathetic network, through propriospinal projections to sympathetic nuclei that are activated in parallel with lumbar locomotor-related interneurons.

### Modulating BP Regulation with SCS: Ascending and Redundant Excitatory Drive to Thoracic SPNs Innervating Blood Vessel Smooth Muscle

As reviewed here, BP can be increased in those with OI by stimulation at multiple rostrocaudal levels (e.g., T6 or T11-L1) and with a variety of stimulation parameters (e.g., 30 vs. 120 Hz). Peripheral nerve stimulation, either with or without muscle contraction in a variety of muscles can also increase systolic and diastolic BP by 10 to 30 mmHg (48–50), similar to increases observed with either TSCS or ESCS. Although either peripheral nerve or spinal electrical stimulation at multiple sites can increase BP, underlying neural mechanisms are not clear. Activation of somatic afferents may be sufficient, given that stimulation over bony prominences of the legs also elicits increased BP in those with OI (49) but it is unknown if stimulation also activates efferent postganglionic neurons in peripheral stimulation and if other spinal structures are activated with central stimulation. Below T1, innervation of blood vessels generally follows a somatotopic organization although vessels of the head and neck require intact connection from sympathetic fibers exiting the cord starting at T1. With vasoconstriction in the splanchnic region mediating increases in BP and its sympathetic innervation from SPNs primarily between T6 and T12, it is interesting that stimulation at more caudal levels (e.g., L1 to S1) normalizes BP regulation, in the absence of leg muscle activation. These findings suggest the presence of as-yet unidentified ascending intraspinal projections to thoracic SPNs, which are discussed further in *Integration within and between Sympathetic and Locomotor Systems at the Spinal Level*. Given the redundant nature of spinal sympathetic neural pathways, with multiple branches of efferent fibers within and between paravertebral ganglia, extensive electrical, and synaptic coupling between preganglionic neurons within and throughout the thoracic intermediolateral (IML) column and multiple points at which sensory body input can influence rates of tonic discharge of postganglionic sympathetic nerves, it is quite likely there will be multiple stimulation strategies capable of influencing a given sympathetic tissue (51–55).

### Modulation of Whole Body Sudomotor Function and Spinal Organization of Sympathetic Input to Sudomotor Tissues

Persons with SCI above T1 are essentially poikilotherms, unable to regulate their body temperature (8, 56–58). The fact that multiple studies reported anecdotally that spinal electrical stimulation could improve cooling sudomotor function, years after SCI, is intriguing and remains to be systematically investigated. Retrograde tracing studies show SPNs innervating cutaneous vasodilators are innervated solely by sympathetic input, are located in the IML, and receive input from cell bodies of the parapyramidal region of the rostroventrolateral medulla [RVLM (59)]. Similar to innervation of smooth muscle of blood vessels mediating increases in BP in humans, rostrocaudal distribution from SPNs to cutaneous vasodilators and sudomotor glands and the ability to sweat in response to heat after SCI follows the dermatomes for injury below T1. For those injured above T1, heat regulation is managed behaviorally, since inaction would result in continuous increases in core temperature while resting in hot environments (60) and can become function limiting or dangerous during upper body exercise in heat (61–64). With SCI at or below ∼T6, those with paraplegia show similar responses to able-bodied controls for similar workloads at 35°C (65).

### Modulating Whole Body Metabolism and Homeostasis: Potential Underlying Neural Mechanisms

Descending “central exercise drive” arising from the hypothalamus was historically thought to mediate catecholamine release from adrenal glands during exercise and circulating catecholamines, which could then act directly on target tissues such as the heart, blood vessel smooth muscle, sweat glands, and fat. However, retrograde tracing demonstrates direct sympathetic innervation to each of these targets from SPNs, beginning at T1 and progressing caudally with a generally somatotopic organization for fat, blood vessels, and sweat glands (Fig. 1). Adrenal gland innervation occurs from T6 to T10. Correspondingly, athletes with injury above T1 have absent or negligible circulating catecholamine and free FA levels during prolonged upper body exercise, including after half-marathons, compared with those with low-level paraplegia (66–71). Together, these factors contribute to the extremely low resting and exercise-based energy expenditure in individuals with cervical level injury, such that even exercising at “high intensity,” based on perceived exertion, for 1 h daily, would result in caloric expenditure considered “sedentary” when compared with neurologically intact sex and age-matched persons (72).

That upper lumbar SCS increased proportion of FA metabolism (up to 8 times) during movement, reduced RPE and improved peak exercise responses as reviewed here supports a role for spinal electrical activation of adrenergic and white adipose tissue (WAT) targets either directly or secondarily to activation of lumbar locomotor-related neural components. Greater endurance, reduction in whole body metabolic substrate use and increased proportion of reliance on fatty acid, rather than glucose oxidation suggests an increase in oxidative motor unit pools and/or increased release and use of fatty acids from fat stores during movement (8, 36). Spinal mechanisms mediating these whole body metabolic adaptations may include changes to proportion of motor units recruited via segmental afferent activation of lumbar locomotor-related interneuron pathways, activation of ascending spinal pathways to thoracic sympathetic centers, and/or recruiting supraspinal autonomic and locomotor-related neural circuitry. Direct sympathetic innervation of WAT (73) suggests capability for direct spinal activation of these tissues although the underlying mechanisms are not well described (74). The observation that there is a whole body “flare-up” in carbohydrate oxidation rate when people walk at speeds just outside of their preferred cadence (75) suggests a strong entrainment between neural circuitry responsible for rhythm-generation and circuits responsible for either selecting and activating specific motor unit pools and possibly also triggering SPN activation of fatty acid release from body fat stores for a given locomotor rhythm. The observation that this relationship can change with training suggests there is significant capability for neural plasticity within circuits that integrate these functions. Whether alterations in whole body metabolism are due simply to a shift in motor pool excitability versus a more widespread systemic activation is unknown but deserves further investigation.

### Spinal Neural Structures Activated by SCS: Parallels between Motor and Sympathetic Systems

Based on human experiments and modeling studies comparing pulsed transcutaneous and epidural stimulation, Minassian and colleagues report similar and multiple spinal neural structures are activated, including dorsal root afferents, motoneurons, intraspinal neurons, and dorsal column fibers (76–81). With increasing intensities of stimulation, the recruitment of dorsal root afferents precedes motor efferents, which precede dorsal column activation due to their proximity and orientation with respect to stimulator cathode, surrounding CSF, and vertebral geometry (82). The order of recruitment of sympathetic afferents and efferents would be expected to be similar, based on their traveling in the same dorsal and ventral roots within the vertebral column. Outside the vertebral column, there is considerable redundancy within and between sympathetic paravertebral ganglia which may enable more widespread activation of sympathetic targets at a given spinal level of either epidural or transcutaneous spinal stimulation. Because of the preferential activation of afferent fibers, in order for transcutaneous delivery of sufficient current amplitude to influence motor outcomes, recent adaptation of stimulators has included a carrier wave implemented so that deeper spinal neural structures can be activated with minimal nociceptive sensory activation (38), although the carrier wave may preferentially activate remaining intact cortical inhibitory pathways (83). It is unknown whether activation of somatic afferents limits activation of sympathetic efferents with TSCS.

In addition to these generalities regarding activation of different spinal structures with either SCS, very specific and individualized stimulation parameters and lead locations are necessary to influence either motor or sympathetic autonomic responses as reviewed here (15, 32) and reported elsewhere (e.g., Refs. 38, 43). At the same time, simply altering the frequency of pulsed stimulation at the same rostrocaudal spinal location can convert one motor behavior into another within the same individual. For example, stimulation at lower frequencies (5–15 Hz) in the rostral lumbar region elicits stance or bilateral extension whereas higher frequencies (25–50 Hz) with similar pulse widths (∼200 µs) elicits stepping within the same individual (10, 84, 85). There are particular peripheral nerve stimulation frequencies thought to specifically activate sympathetic target tissues. For example, intraneural stimulation of leg cutaneous nerves at 2 Hz in able-bodied participants increased FA release by 47% from leg WAT stores, while not affecting circulating FFA or catecholamines (86). It is unknown if stimulation at different frequencies would preferentially activate specific and distinct sympathetic targets. Work done by Sato and colleagues in cat and rat suggests that this may be the case since the appearance of sympathetic reflexes recorded from lumbar white rami depends upon the somatic nerve stimulation rate and train characteristics (53, 54, 87).

### Parallels in “State-Dependency” of Locomotor and Exercise-Related Sympathetic Spinal Responses Evoked by SCS

Spinal neural circuitry generating and coordinating locomotor rhythms is redundant and widely distributed throughout the spinal cord (88–93). For motor systems, there may be multiple subthreshold yet excitatory effects of SCS, based on observations that the particular motor output elicited during stimulation can be influenced by sensory input and body positioning (43). Specifically, mainly flexor responses are elicited with spinal stimulation when supine whereas the same stimulation elicits predominantly extensor discharge when stimulation is delivered while upright (94). As reviewed here, SCS at the same spinal location with similar stimulation parameters can mediate opposite effects on BP regulation. TSCS at T7/T8 reduced the rise in BP induced by rectal stimulation (23) whereas stimulation at T7/T8 increased BP in response to an orthostatic challenge (20). Sachdeva et al. (23) proposed that their stimulation preferentially activated large diameter afferents that effectively gated out the nociceptors activated by digital stimulation. It may also be that SCS evoked sympathetic responses are influenced by other sensory inputs received at the time of stimulation, such that an increase in BP occurs in some conditions and a decrease in other conditions, depending upon the combination of afferent, descending and intraspinal sources of excitatory input to SPNs mediating vasomotor constriction.

Descending serotonergic systems activated from the mesencephalic locomotor region (MLR) and arising from the brainstem elicit “state dependent” changes in spinal premotor neurons that facilitate rhythmic motor activity, including voltage threshold hyperpolarization, plateau potentials, persistent inward currents, and may be involved in presynaptic inhibition of hindlimb afferents during locomotion (95–100). These changes often precede the onset of locomotor discharge in response to stimulation of either the MLR or brainstem medullary locomotor structures (8, 101). Thus, simply changing the spinal neural state to a more excitable level would allow for phase and activity-dependent sensory inputs to act upon a primed spinal cord, including lumbar interneurons and motoneurons, enabling various motor behaviors. These factors likely contribute to the emergence of lower and upper limb motor activity with SCS and may contribute to autonomic sympathetically mediated functions as well. The fact that thoracic sympathetic nuclei receive dense innervation from descending serotonergic fibers arising from the brainstem (102) supports the concept that locomotor and sympathetic metabolic and homeostatic neural circuitry are simultaneously activated or modulated by descending commands from brainstem regions. There is emerging evidence (discussed further in *Integration within and between Locomotor and Sympathetic Systems at the Level of the Brainstem*) that integration between descending locomotor and homeostatic commands may indeed occur at the level of the rostroventrolateral (RVLM) medulla, and likely involves multiple neurotransmitter systems.

Stimulation at specific phases of the step cycle and at different spinal levels indicates that precisely timed phasic input to spinal neural structures can enable more refined and precise lower limb motor output than tonic stimulation alone (44). These findings are similar to studies in cat and rat, in which afferent input to different limb joints can have a greater or lesser effect on limb muscle output, depending upon the position of the limb and/or phase of the step cycle in which it is received (103–106). It is unknown and probably unlikely that metabolic and homeostatic sympathetic targets would need to be activated in a similar phase-dependent manner as needed for overground motor function. However, somatic afferents activate sympathetic spinal reflexes and likely do so during rhythmic locomotor activities. For example, stimulation of somatic sensory limb or trunk afferents induce reflex increases in discharge rates of L2 white rami in intact and spinal animals (52, 54). Thus, activation of leg afferents can provide excitatory input to sympathetic preganglionic fibers, in addition to their effects on lumbar locomotor-related interneurons. These reflex responses can be elicited in both lumbar and cervical sympathetic white rami, indicating that afferent input from leg afferents provides widespread ascending input to thoracic sympathetic nuclei (87). The “pressor effect” of increasing BP by lower limb afferent activation may contribute to some of the observations reported here (51, 107), but there are likely additional mechanisms. For example, somatic nonnociceptive afferent input can either increase or decrease metabolic and homeostatic sympathetic responses depending on the direction of cutaneous activation. Specifically, brushing an anaesthetized animal’s fur in its natural direction reduces adrenal sympathetic nerve discharge rates and catecholamine release from adrenal veins whereas brushing against its fur grain increases them (53). Furthermore, this response is absent or reversed after acute spinal transection at C1/2, such that multisegmental distant cutaneous-evoked events are absent (e.g., stroking fur of the shoulder no longer reduces catecholamine release) and local segmental responses are reversed (e.g., stroking stomach fur now increases catecholamine release from adrenal gland) (53). Finally, these somatic afferent-evoked sympathetic reflexes display a period of hyporeflexia immediately after spinal transection, followed by a delayed emergence of hyper- or dysreflexic responses (52). Thus, similar to motor reflexes (e.g., Babinsky sign), or other autonomic functions such as afferent-induced bladder hyperreflexia, spinal transection removes descending communication from supraspinal centers and alters these sympathetic spinal-evoked metabolic and homeostatic reflexes. In relation to persons with SCI, these findings suggest there may be significant spinal sympathetic neural circuitry that can be activated or manipulated by SCS either alone or in concert with locomotor-related activation.

### Direct Current and Pulsed SCS Could Alter Exercise-Related Sympathetic Activity

All studies reported here used pulsed stimulation with the exception of Shelyakin et al. (29), who used direct current stimulation and reported an increase in HR of participants in addition to improvements in lower limb motor control that they attributed to activation of thoracic paravertebral ganglia. Other studies in those without injury report improved motor function with direct current lumbar stimulation (108). As investigated and reviewed by Jankowska and colleagues, direct current stimulation may be an alternate or supplementary means to induce long-lasting excitation of spinal neural structures, particularly neurons with fibers traveling in the dorsal columns (109–112). We did not find any studies directly comparing effects of pulsed versus direct current (DC) stimulation. Our focus in this review was acute responses to SCS only. Given the potential for direct current to modulate long-lasting excitation of spinal neural structures, it is of interest to determine if pulsed and DC SCS are each similarly capable of facilitating acute and longer-term plasticity-dependent responses within locomotor and sympathetic systems.

### Integration within and between Locomotor and Sympathetic Systems at the Level of the Brainstem

Retrograde labeling using pseudorabies virus injection (PRV) and electrophysiological studies have revealed a consistent pattern of organization for descending input to spinal neurons innervating adrenal, cardiovascular, thermoregulatory, adipose, and skeletal muscle tissues. Performed in different studies, after injection of PRV into these tissues, labeling is observed in SPNs at the spinal levels indicated in Fig. 1. With longer survival times, PRV-labeling is seen in neurons within the same spinal segments in contralateral sympathetic nuclei and in neurons in the parapyramidal region of the RVLM. In addition, when different tissues are injected with different PRV dyes in the same animal, such as into WAT and muscle or adrenal glands and WAT, colabeling of neurons in the RVLM is seen in subsets of neurons labeled from each tissue [(8, 51, 59, 113–117) and reviewed in Ref. 8]. These studies reveal a similar organization of descending input from RVLM to thoracic sympathetic nuclei and suggest redundancy and overlap with respect to input from brainstem regions regulating control of these different metabolic and homeostatic tissues. Furthermore, each of these systems receives integration at the level of the RVLM before a descending command signal provides input to thoracic IML to adjust activity levels in SPNs innervating these key target tissues (reviewed in Ref. 116). This region has been proposed as a key integration site and descending command center for cardiovascular control (51, 107), thermoregulation (118–121), and regulation of lipolysis from WAT (74). This region of the RVLM is also the final relay for descending locomotor command signals elicited by stimulation of the MLR (101). Chemogenetic activation of serotonin neurons in the parapyramidal region elicits hindlimb locomotion and simultaneously increases BP, which precedes and outlasts each bout of locomotion in a genetic decerebrate rat model (122). Therefore, a unifying conceptual framework emerges in which this region of the RVLM serves as a key integration center, not just for each of these systems separately, but also for integrating descending locomotor command signals with descending sympathetic drive to homeostatic and metabolic tissues (8). This framework proposes that this region serves to either enable or disable rhythmic locomotor commands, depending upon body homeostasis and presence of sufficient resources to maintain ongoing rhythmic motor activity (i.e., exercise).

**Figure 4.**
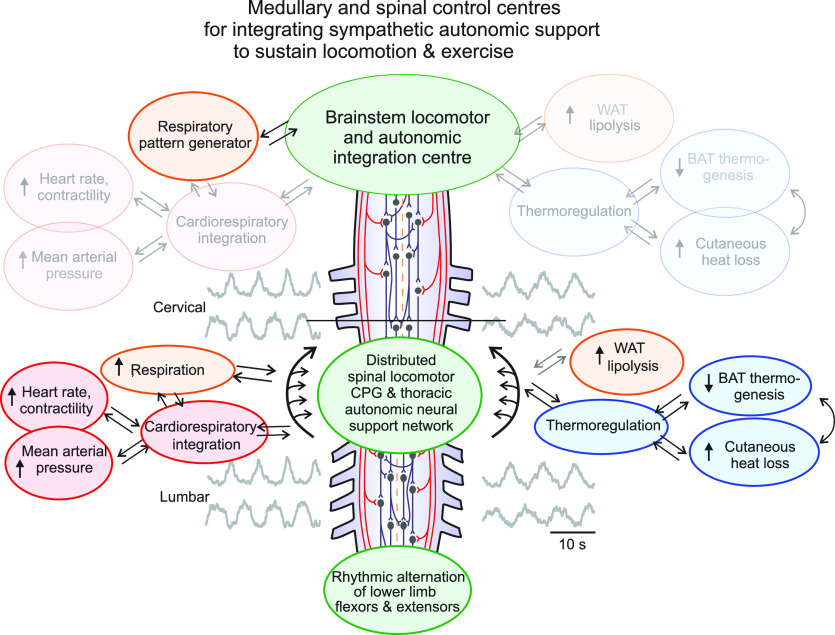
Schematic outlining how spinal autonomic functions needed to support movement and exercise may be activated in the absence of descending regulatory control after SCI. Locomotor and autonomic integration and descending command neurons in the rostroventral medulla mediate activation of spinal locomotor and sympathetic autonomic functions needed to support movement. Normally, descending activation of locomotor circuitry occurs simultaneously with increased activation of cardiovascular, respiratory, temperature-regulating, and whole body metabolic energy stores (WAT). After SCI at or above T1, this integrated descending command system is completely disrupted (nonfunctional systems faded out) and signals cannot reach spinal sympathetic systems located between T1 and L2, and therefore cannot increase neural activation of target tissues. Spinal electrical stimulation studies in humans and multiple animal-based autonomic studies reveals extensive capacity for spinal neural mechanisms to mediate increased activation of these autonomic tissues. Further, these studies suggest an intraspinal ascending drive from lumbar locomotor related neurons to thoracic spinal autonomic centers. Although the ability to influence these systems with spinal electrical stimulation is demonstrated, the underlying neural pathways and mechanisms remain unknown. Modified with permission from Cowley (8).

In this schema, activation of locomotor activity occurs simultaneously with increased sympathetic output to needed metabolic and homeostatic support systems that increase BP, HR, tissue cooling, and fat mobilization during movement. Normally, descending commands for activation of rhythmic motor activity or exercise would be integrated with activation of descending regulatory commands to thermoregulatory, cardiovascular and metabolic tissues at the brainstem level, as well as with sensory input from these tissues and organs. This framework also posits that there are intraspinal connections between lumbar locomotor-related neurons and thoracic metabolic and homeostatic sympathetic neural circuitry. Normally descending commands from the brainstem would regulate the activity of these sympathetic tissues. However, after complete SCI, these intraspinal connections would operate in the absence of any descending input from supraspinal regions (Fig. 4). This framework may help explain why SCS to activate lower limb locomotor activity also improves the exercise-related sympathetic functions reviewed here. Specifically, SCS activates spinal motor circuitry which is integrated with activation of thoracic sympathetic neural circuitry activating cardiovascular, thermoregulatory and metabolic tissues at multiple spinal levels.

### Integration within and between Sympathetic and Locomotor Systems at the Spinal Level

There are likely ascending intraspinal projections from lumbar locomotor-related interneurons onto thoracic sympathetic neurons, and these may play a role in the generation and maintenance of movement and exercise after SCI (Fig. 4). The rationale for this is based upon multiple lines of evidence. For example, spinal redundancy of pathways that contribute to or elicit lumbar locomotor activity has been well established from in vitro locomotor studies (89–92, 123). There are long interconnections between cervical and lumbar regions involved in coordinating upper and lower limb motor activity (124), some of which may include long ascending excitatory fibers (125) but whether they provide collateral input to thoracic IML neurons is unknown. Ascending projections from lumbar and thoracic levels exist and can entrain or drive upper limb function during locomotion (126–130). In contrast, the ability for descending projections from the upper limb to drive lower limb motor activity is less powerful (127, 131). In vitro studies in neonatal rat have also demonstrated that activation of sacral neural circuitry can activate lumbar locomotor circuitry and may preferentially elicit stance or bilateral extensor discharge compared to stimulation at more rostral lumbar sites, demonstrating a strong ascending gradient of excitability in motor systems (132–137). This is consistent with extensor-dominated discharge with more caudal stimulation in humans as well (43).

In addition, particular spinal segments are critical for mediating or integrating locomotor command signals. Specifically, locomotor activity will persist and remain coordinated between left and right in response to brainstem electrical stimulation after extensive midsagittal lesions throughout the rostrocaudal extent of the spinal cord, provided two to three contiguous segments remain intact, and if these bridging segments include T13 and/or L1 (123). Thus, commissural projections in this region are critical for generating locomotor activity in response to descending command signals arising from the brainstem. These segments are also critical when locomotor activity is induced in the isolated spinal cord by bath-applied serotonin and N-methyl-d-aspartate (123). Whether neurons in these segments, collaterals traveling through these segments and/or ascending projections from more caudal regions also provide input to thoracic sympathetic neurons during movement is unknown and warrants investigation.

Although many questions remain, the picture that is emerging suggests multiple mechanisms mediate integration between locomotor and sympathetic systems within the spinal cord. Activation of cutaneous and proprioceptive muscle afferents elicit reflex interneuron responses that increase firing rates of SPNs innervating cardiovascular, sudomotor and metabolic tissues at the local segmental level and at more distant intraspinal levels (54). These reflex responses act upon basal levels of firing within SPNs. When neurologically intact, the spinal sympathetic reflex responses are smaller and/or modulated by somato-sympathetic reflex centers in the medulla (54). In the absence of descending input from the brainstem, spinal somato-sympathetic reflexes dominate and may be altered in direction or amplitude of response (54). Spinal interneurons that organize these reflex responses have not been identified and how this interneuron communication changes in the absence of descending input from the brainstem is unknown. Propriospinal locomotor rhythm-generating circuitry within and throughout the spinal cord is redundantly distributed and likely integrated with neurons in the IML and other sympathetic nuclei at each thoracic segmental level throughout the thoracic spinal cord. Subpopulations of interneurons mediating such integration have not been identified. There may also be cutaneous or muscle afferents from particular limbs, muscles or skin locations that more effectively elicit sympathetic reflex responses. The associated subpopulations of spinal neurons mediating these reflex responses have not been identified. Another contributing mechanism may be the recruitment of additional or distinct subpopulations of interneurons that become active during locomotion and alter the direction or amplitude of these sympathetic responses, as has been described to occur within the motor system. Specifically, group I extensor afferent-evoked oligosynaptic inhibitory reflex motor responses at rest are converted to disynaptic excitatory responses during locomotion elicited by electrical stimulation of the mesencephalic locomotor region, and this excitation is thought to be mediated by a subpopulation of excitatory interneurons in the intermediate laminae in midcaudal L7 that are not operational in the nonlocomoting preparation (138, 139). Specific spinal segments containing subpopulations of interneurons that more effectively mediate excitatory input to thoracic SPNs during locomotion may exist but have not been identified. There may be particular spinal segments containing neurons or axon collaterals that are key for integrating or relaying activity in locomotor CPG neurons to sympathetic nuclei that in turn increase SPN output to these tissues. Future research identifying intraspinal neurons linking rhythm-generating locomotor circuitry and sympathetic nuclei, and understanding the neurochemical mechanisms integrating their respective activities will be important for identifying means to specifically activate these sympathetic targets in persons with SCI.

### Conclusions

SCS has the capacity to improve motor and autonomic functions in those living with SCI. Clinical research demonstrates potential for TSCS and/or ESCS to improve sympathetic metabolic and homeostatic functions needed for exercise. These studies are limited in terms of being mainly observational, uncontrolled case reports or series, but show promise and highlight the need to better understand the spinal neural mechanisms mediating these responses. More systematic and targeted clinical examination of spinal stimulation to influence these exercise-related autonomic outcome measures is warranted. Optimizing the ability for neuromodulation to target these specific functions will also require a better understanding of how intraspinal neural mechanisms facilitating motor functions are integrated with sympathetic activation of these tissues during movement and exercise. Ultimately, application of neuromodulation strategies targeting these functions will provide means to maintain body homeostasis at rest under different environmental conditions and increase exercise capacity, and thereby prevent or delay onset of metabolic and vascular diseases common after SCI.

## GRANTS

This work was supported by the Natural Sciences and Engineering Research Council of Canada (NSERC): RGPIN-2015-04810, Research Manitoba, Canada Research Chairs.

## DISCLOSURES

No conflicts of interest, financial or otherwise, are declared by the author(s).

## AUTHOR CONTRIBUTIONS

K.C.C. conceived and designed research; S.F. and J.G. performed experiments; S.F., J.G., and K.C.C. analyzed data; S.F. and K.C.C. interpreted results of experiments; S.F., J.G., and K.C.C. prepared figures; S.F. and K.C.C. drafted manuscript; J.G. and K.C.C. edited and revised manuscript; S.F., J.G., and K.C.C. approved final version of manuscript.

**Table A1.** SCS Clinical Trials on ClinicalTrials.gov April 28, 2022

Study Name	Type of Stimulation	Information Provided by (Location)	Status	Estimated/Actual Participants (*n*)	Primary Outcomes	NCT
Restoring Hemodynamic Stability Using Targeted Epidural Spinal Stimulation Following SCI (STIMO HEMO)	ESCS	Bloch, Jocelyne (Center Hospitalier Universitaire Vaudois)	R	8	BP during OH tilt table test	NCT04994886
Non-Invasive Spinal Cord Stimulation After Injury	TSCS	Ovechkin, Alex (University of Louisville)	R	36	V̇o_2_	NCT03998527
Transformation of Paralysis to Stepping	TSCS	Ovechkin, Alex (University of Louisville)	R	15	Body temp, BP, HR, respiration rate, DEXA scan	NCT04105114
Epidural Stimulation After Neurologic Damage (E-STAND)	ESCS	University of Minnesota	R	100	BP, cerebral blood flow during tilt table test	NCT03026816
Standing, Stepping and Voluntary Movement Spinal Cord Epidural Stimulation	ESCS	Harkema, Susan (University of Louisville)	A, NR	16	RMR after 1 y of treatment	NCT04123847
Recovery of Cardiovascular Function With Epidural Stimulation After Human SCI	ESCS	Harkema, Susan (University of Louisville)	C	4	CV function	NCT02037620
Task-specific Epidural Stimulation Study (TS EPI)	ESCS	Harkema, Susan (University of Louisville)	R	36	CV function	NCT03364660
SCI Epidural Stimulation	ESCS	Zhao, Kristin (Mayo Clinic)	C	2	Thermoregulation, body fat, bone density, lean mass	NCT02592668
Epidural Stimulation After SCI (ESL-SCI)	ESCS	McGuire Research Institute	NYR	5	O_2_ consumption and BP (sitting, standing, walking), body composition (before and after training)	NCT04105296
Neuromodulation Techniques After SCI	EKSO suit + ESCSEKSO suit + TSCS	Virginia Office of Research Development	R	10	Fat mass and fat free mass (every 3 months), O_2_ consumption (sitting, standing, walking), fasting lipid profile	NCT04241250
Effects of Transcutaneous Spinal Cord Stimulation in SCI*	TSCS	Korupolu, Radha (The University of Texas Health Science Center)	C	15	BP, HR	NCT03249454
Spinal Cord Stimulation and Autonomic Response in People With SCI	TSCS	Phillips, Aaron (University of Calgary)	NYR	46	BP (continuous and episodic), cerebral blood flow, HR, sympathetic skin responses, tilt table OH challenge, cerebrovascular structural changes, catecholamine levels, AD QOL questionnaire	NCT03924388
Neuromodulation to Improve Respiratory Function in Cervical SCI	ESCS	Lu, Daniel (UCLA)	R	15	BP (beginning and end of each stimulation session for safety)	NCT04883463
Spinal Cord Stimulation to Augment Activity Based Therapy	TSCS	Shepherd Center (Atlanta, GA)	C	18	HR during training, RPE	NCT03240601
Neuromodulation: Bladder Bowel and Sexual Function in SCI	TSCS	Krassioukov, Andrei (University of British Columbia)	NYR	40	BP (continuous for identifying AD events)	NCT04604951
Improving Bowel Function and Quality of Life After SCI	ESCS	Herrity, April (University of Louisville)	R	36	Ambulatory BP and HR	NCT03949660
Recovery of Bladder and Sexual Function After Human SCI	ESCS	Hubscher, Charles (University of Louisville)	R	70	Continuous BP monitoring	NCT04193709
Transcutaneous Spinal Cord Neuromodulation to Normalize Autonomic Phenotypes	TSCS	Solinsky, Ryan (Spaulding Rehabilitation Hospital)	NYR	10	Muscle sympathetic nerve activity, HR, BP, galvanic skin response, AD and OH questionnaires	NCT04858178

A, active; BP, blood pressure; C, completed; CV, cardiovascular; DBP, diastolic blood pressure; ESCS, epidural spinal cord stimulation; NR, not recruiting; NYR, not yet recruiting; OH, orthostatic hypotension; R, recruiting; SBP, systolic blood pressure; SCI, spinal cord injury; TSCS, transcutaneous spinal cord stimulation.

## APPENDIX

Current studies investing the use of transcutaneous or epidural spinal stimulation on sympathetic functions that support movement and exercise are shown in Table A1. At submission, 18 studies are listed on clinicaltrials.gov investigating effect(s) of spinal electrical stimulation on these sympathetically mediated functions.
